# Phase-Change Materials as Cryo-Shock Absorbers in Rigid Polyurethane Cryogenic Insulation Foams

**DOI:** 10.3390/polym17060729

**Published:** 2025-03-10

**Authors:** Laima Vevere, Beatrise Sture-Skela, Vladimir Yakushin, Pavel Němeček, Hynek Beneš, Ugis Cabulis

**Affiliations:** 1 Latvian State Institute of Wood Chemistry, Dzerbenes Str. 27, LV 1006 Riga, Latvia; laima.vevere@kki.lv (L.V.); beatrise.sture@kki.lv (B.S.-S.); vladaimir.yakushin@kki.lv (V.Y.); 2 Institute of Macromolecular Chemistry, Czech Academy of Sciences, Heyrovskeho nam. 2, 162 00 Prague 6, Czech Republicbenesh@imc.cas.cz (H.B.)

**Keywords:** phase-change materials, cryogenic insulation, rigid polyurethane foams, mechanical properties, adhesion

## Abstract

This study investigates the effects of microencapsulated phase-change materials (PCMs) on the density and thermal conductivity of rigid polyurethane (PU) foams, alongside their mechanical properties. Introducing PCMs into the foam composition results in increased viscosity, complicating the mixing of polyol and isocyanate components. This viscosity increase can slow the foaming rate and subsequently raise the foam density, as observed in both poured and sprayed rigid PU foams containing 5% and 10% PCM, leading to density increases of up to 9%. Despite these slight density changes, the thermal conductivity remained relatively stable due to the preservation of the foam’s closed-cell structure. The mechanical evaluation revealed a decrease in compressive and tensile strength with a higher PCM content attributed to defects arising in the foam’s cellular architecture. However, adhesive strength to aluminum substrates improved, particularly with 5% PCM, possibly due to a more consistent foam structure during the slower foaming process. Differential scanning calorimetry and a dynamic mechanical analysis indicated that the incorporation of PCM increased the glass transition temperature and affected the foam’s mechanical properties. This research underscores the potential of microencapsulated PCMs to enhance the functionality of rigid PU foams while needing careful consideration of their concentration to avoid compromising the structural integrity.

## 1. Introduction

The demand for advanced insulation materials in cryogenic applications has seen a rapid rise, driven by the need for the efficient storage and transportation of liquefied gases, such as hydrogen (boiling point −253 °C), nitrogen (b.p. −196 °C), oxygen (b.p. −183 °C), and natural gas (b.p. −161 °C). Among the various insulation options, rigid polyurethane (PU) foams have emerged as a widely used choice due to their excellent thermal properties, lightweight nature, and versatility [1]. Owing to their notable mechanical characteristics, rigid PU foams have also been applied to several cryogenic insulation appliances, where the exploitation temperature drops to 120 K (−153 °C) and lower [2]. The demand for different cryogenic insulation tanks is growing because of their varied usability. In the aircraft and space industry, liquefied gases are commonly used as fuel; therefore, an appropriately insulated cryogenic tank is a must so that the unnecessary evaporation of gases can be prevented. Since the European Union is determined to reach climate neutrality by 2050, multiple economic sectors, including aviation, must decarbonize, which means that one of the conditions will be using liquefied hydrogen and/or other gases as fuel [3,4]. Also, liquefied natural gas is more frequently used for domestic and industrial on-ground processes [5]. For LNG containers, rigid PU and polyisocyanurate foams reinforced with glass and aramid fibers, cellulose nanocrystals, etc., in order to increase mechanical stability, have been widely used [6,7,8,9]. Rigid PU foams used in such appliances must withstand tremendous mechanical stresses and temperature differences. Another advantage of rigid PU foam is its ability to be sprayed on different surfaces—it has excellent adhesion not only with steel and aluminum from which containers are usually made but also with plywood and other materials, which are usually part of the insulation layer [10]. PU foams are, of course, a very suitable material for cryogenic insulation and are widely used, but sometimes, they lose out to multi-layer insulation in terms of their properties [11], so new methods need to be found to increase the efficiency of PU cryogenic insulation, thus making it more attractive to end users.

The Green Deal in chemistry not only encourages the usage of environmentally friendly fuels but also enhances the attention to physical blowing agents used in rigid PU foam production. In recent years, hydrochlorofluoroolefins (HCFOs) have taken place as the most promising blowing agents since their ozone depletion potential and global warming potential are close to zero, which correlates with EU regulation No. 517/2014 [12]. Yakushin et al. have studied the impact of more eco-friendly blowing agents and catalysts on rigid PU foams’ properties in several articles [13,14]; a decrease in the coefficient of thermal conductivity was observed, which is a significant characteristic of rigid PU foams used for cryogenic insulation. 

Polyols for the production of rigid PU foams can be easily synthesized from vegetable oils and different biomass products, for example, rapeseed oil [15,16,17], coconut oil [17,18,19,20], soybean oil [21,22,23], tall oil [24], lignin [25], suberinic acids [24], and succinic acids [26], etc., therefore replacing petrochemical-based polyols. A Life Cycle Assessment (LCA) has been applied to quantify the environmental impacts of bio-based polyols suitable for PU production. LCAs show that the environmental performance of bio-polyols is not unidirectional. Fridrihsone et al. (2020) reported that rapeseed-oil-based polyol has better environmental performance in 8 out of 18 ReCiPe midpoint impact categories and a lower cumulative energy demand compared to its petrochemical counterpart [27]. Staccioli et al. (2024) compared lignin-based polyols with petrochemical alternatives and highlighted that [28] lignin-based polyols demonstrate superior environmental performance under specific conditions, such as the use of bio-based solvents and an appropriate energy mix when compared to their petrochemical counterparts. The introduction of fatty acids obtained from biomass increases the sustainable material content in rigid PU foams, which makes them more appealing to both manufacturers and customers. It is also important to consider whether raw materials for the chemical industry do not compete with food and feed production. In our case, this is clearly not the case, because we use a 2nd generation raw material—tall oil, because its use is also promoted in various ways for fuel production [29].

However, as rigid PU foams are exposed to extremely low temperatures, they can experience thermal stress, leading to performance degradation or structural failure. To mitigate such challenges, the integration of phase-change materials (PCMs) has garnered significant attention [30,31,32]. Cryogenic insulation can also be used for cold storage, but temperatures only slightly below zero are used in this case.

PCMs, which absorb and release energy equivalent to their latent heat during phase transitions, offer the potential to enhance the thermal stability of rigid PU foam cryogenic insulation [33,34,35]. PCMs are often encapsulated in polymeric shells in order to (a) ensure that larger particles of PCM are not damaged during the exploitation of the composite, (b) control energy release, and (c) increase the PCM surface [36,37,38,39,40]. PCMs are often used for thermal energy storage, where a mismatch between the production and use of energy occurs, for example, generating energy from solar panels [26]. Producing composites where different materials (PU, concrete, etc.) are filled with PCM microcapsules can improve the insulation characteristics and thermal storage [41,42,43,44,45]. In [33], it was observed that rigid PU foam–PCM composites (mesoporous silica and tight silica microshells were used as PCM) showed a smaller cell size compared to pure rigid PU foam, which resulted in a slightly increased density, but reduced cell size, induced higher compressive strength by at least 27% and lower thermal conductivity; therefore, the material could be considered an appropriate thermal insulator for different appliances. It has also been observed that the addition of PU–PCM composites in an asphalt binder improves its high-temperature stability, anti-aging properties, and storage stability [46]. Galvagnini et al. added microencapsulated paraffin to rigid PU foam up to 50% of the composite total mass, but unfortunately, the addition of PCM led to the formation of an open cell morphology, which decreased the flexural strength, and no significant improvement in the overall properties of the composite was observed [39]. In [47], nanosized PCMs (n-octadecane encapsulated in silica shell) were added to rigid PU foam, and the composites exhibited a closed-cell structure. In this study, the thermoregulation of composite samples was measured with a thermal camera, and it was observed that the temperature of composites increased slower than that of pure rigid PU foam, which can be explained due to the melting of encapsulated n-octadecane, therefore ensuring that this composite can be used for different appliances where thermal regulation takes place. Similar conclusions were made in [48], where the ability to control temperature was observed near the phase-transition temperature of n-octadecane (the PCM was prepared as n-octadecane encapsulated in sodium silicate shells). 

Zhai et al. obtained composites from rigid PU foam and microencapsulated PCM (Nano-CuO and CNTs polymers). The investigation showed that composites had perfect cycling stability and phase-change enthalpy, which makes this composite suitable for cold storage transportation systems, for example, vaccine transportation [49]. Cold storage transportation can also be used in the food industry to keep products fresh for longer, therefore ensuring their quality and limiting food waste. A cold storage van prototype insulated with rigid PU foams filled with n-tetradecane microencapsulated in poly(methyl methacrylate-co-methacrylic acid) was created and cooled to −15 °C [50]. During the warming process (up to +15 °C), the temperature was measured, and it was observed that the temperature of rigid PU foams containing PCM did not increase as steadily as that of pure rigid PU foams; therefore, the composite can be used to maintain a specific temperature for an extended period of time enhancing the temperature buffer characteristic. This, of course, is an excellent characteristic for cryogenic insulation material, but in this case, PCM had to be added at a high concentration—up to 30% of the total composite mass. 

In [51], microencapsulated PCM was cryogenically conditioned (at liquid nitrogen temperature −196 °C), and it was observed that cyclic cryogenic conditioning resulted in PCMs with smooth surfaces, and no cracks and/or fractures were present, which could help to produce composites suitable for cryogenic insulation. Also, it was observed that cryogenically conditioned PCMs showed a higher onset thermal decomposition temperature compared to PCMs. Even though rigid PU foams are being extensively used for insulation, some decrease the mechanical properties, like the flexural strength, after the addition of phase-change materials (PCMs) [35]; therefore, research for novel methods to increase the efficiency of cryogenic insulation must be continued.

Our scientific group has many years of experience in the development of cryogenic insulation materials, including cooperating with the space industry [52]. The most important characteristics of good and high-quality cryogenic insulation are not only the mechanical strength and thermal conductivity coefficient but also such properties that are not studied for conventional insulation, such as the adhesion strength to the metal (usually aluminum or stainless steel) surface of the tank, where the liquefied gases are filled, before and after cryo-shock, that is, before and after filling with the cryogenic liquid. The second parameter that determines the quality of cryogenic insulation is the safety coefficient, which characterizes the ability of the insulation material to maintain elasticity at extremely low temperatures and compensate for the different thermal expansion coefficients of foam and metal [53].

This paper explores the innovative use of PCMs in rigid PU foams, particularly for cryogenic insulation, analyzing their role in improving the cryogenic performance, mechanical durability, and overall insulation efficiency. By leveraging the unique thermal and mechanical properties of PCMs, this approach promises to push the boundaries of cryogenic insulation technology, ensuring safer and more reliable operations in extreme environments. 

## 2. Materials and Methods

### 2.1. Materials

For rigid PU foams, the following materials were used: polyols from the epoxidized tall oil fatty acids ETOFA-DEG and ETOFA-DEOA polyols were synthesized in the Latvian State Institute of Wood Chemistry, NEO 240 (Neo Group, Klaipeda, Lithuania), diethylene glycol (Chempur, Germany), the flame retardant tris(2-chloroisopropyl)phosphate (TCPP) (Albermarle, Belgium), the blowing agents Opteon 1100 (The Chemours Company FC, Dordrecht, The Netherlands), the catalysts Polycat^®^203 (Evonik, Essen, Germany), Polycat^®^206 (Evonik, Essen, Germany), Dabco^®^MB20 (Evonik, Essen, Germany), the surfactant Tegostab^®^ B 84711 (Evonik, Essen, Germany), polymeric 4,4-methylene diphenyl isocyanate (pMDI) (Desmodur^®^ 44V20L from Covestro AG, Leverkusen, Germany), and the encapsulated phase change material Crodatherm ME 29P—PW—(MW) (CRODA NORDICA AB, Limhamn, Sweden). Formulations of rigid PU foams are shown in Table 1.

### 2.2. Preparation of Rigid PU Foams

Using the pouring method, rigid PU foams were produced at room temperature (around 22 °C). After a minute of mixing polyols, catalysts, surfactants, flame retardants, and blowing agents in the appropriate amounts, isocyanate was added in the appropriate amounts and stirred for ten seconds. An open-top mold was filled with everything. For a full day, the foam remained at room temperature. The foam was taken out of the mold and cut into the appropriate samples the next day. Cup tests were prepared in the same way, only after the mixing cup was placed under the Universal Foam Qualification System FOAMAT (Format Messtechnik GmbH, Karlsruhe, Germany) ultrasound sensor, and the rigid PU foam growth was measured. 

Spray foam was prepared using the high-pressure spraying machine Glascraft VR (Graco Inc., Minneapolis, MN, USA). During spraying component A and B, the temperature was 45 °C and the line pressure was 170−180 bar. Rigid PU foams were sprayed on 4 mm-thick aluminum sheets coated with a wax-based release agent in one pass. In addition, the foam was sprayed on small aluminum plates (40 × 40 mm) for an adhesion test.

### 2.3. Apparent Density 

The ISO 845:2006 standard [54] was used to test the obtained rigid PU foams’ apparent density.

### 2.4. Apparent Viscosity

The rheological measurements were performed using an Anton Paar modular compact rheometer MCR 92 (Anton Paar, Graz, Austria), with a cone-plate measuring system and a gap of 48 μm. The apparent viscosity of the polyols was measured at 25 °C, and the shear rate was 50 s^−1^, using standard flow curve measurements and shear rate sweep from 0.1 s^−1^ to 100 s^−1^.

### 2.5. Coefficient of Thermal Conductivity 

The Fox 200 (TA Instruments-Waters LLC, New Castle, DE, USA) was used to determine the thermal conductivity coefficient. The test was carried out in compliance with ISO 8301:1991 [55]. Three 20 × 20 × 5 cm samples were inserted between two plates, one of which was at 20 °C and the other was at 0 °C. At a mean temperature of +10 °C, the thermal conductivity coefficient was obtained. 

### 2.6. Compression Strength 

Zwick/Roell Z010 (10 kN) (Zwick Roell, Ulm, Germany) static materials testing equipment combined with a 1 kN force cell was used to perform compression tests at room temperature in accordance with ISO 844:2021 [56]. Cylindrical samples of 20 mm in diameter and 22 mm in height were utilized for the compression tests. The tests were conducted in two directions: parallel (Z) and perpendicular (X) to the foam rise. Twelve samples in all, six in each direction, were used. 

Compression tests were performed using a Zwick/Roell Z100 (100 kN) +1 kN (Zwick Roell, Ulm, Germany) force cell and a cryostat at a liquid nitrogen (LN2) temperature in accordance with ISO 844:2021 [56]. Cylinders that ranged in size from 20 mm in diameter to 22 mm in height were used for the tests. 

### 2.7. Tensile Strength 

Testing was performed using the Zwick/Roell Z100 (100 kN) +1 kN force cell with a cryostat, in accordance with ISO 1926:2009 [57], for Young’s modulus and the tensile strength at a liquid nitrogen temperature. Ring-type samples (width: 13 mm; inner diameter: 43 mm; outer diameter: 53 mm) were employed, and six samples were evaluated in parallel. According to ASTM D 2290 [58], the rings were cut out in-plane perpendicular to the direction of the foam rise.

### 2.8. Adhesion

Tensile strength tests were used to determine the rigid PU foam’s adhesion to an aluminum plate. Using a 1 kN force cell and the Zwick/Roell Z010 (10 kN) static materials testing equipment, testing was carried out in accordance with EN 1607:2013 [59]. The foam material and aluminum plates, each with a total thickness of 20 mm, were bonded using PU adhesive between the two sample holders. Sixteen samples in all were analyzed, eight of which were not submerged in liquid nitrogen and eight of which were. In order to evaluate the effect of extreme temperature conditions on the adhesion qualities of the foam–aluminum bond, adhesion strength tests were performed both before and after immersion in nitrogen for one hour (cryo-shock).

### 2.9. Differential Scanning Calorimetry

The differential scanning calorimetry (DSC) was performed using the Mettler Toledo DSC 823e (Mettler Toledo, Greifensee, Switzerland). PCM samples were not pretreated before testing. Prior to testing, rigid PU samples were pulverized in a cryogenic ball mill. Aluminum crucibles were utilized for the test, and the tested samples weighed roughly 7 mg. After being heated from 25 °C to 180 °C (10 °C/min), the rigid PU foam sample was cooled from 180 °C to −100 °C (10 °C/min) and then heated once more to 180 °C (10 °C/min).

The PCM cycling stability was checked by heating rigid PU foams containing PCM to 90 °C and cooling to −30 °C. Heating/cooling cycles were repeated 50 times.

### 2.10. Dynamic Mechanical Analysis

The ARES G2 (TA Instruments) was used for the dynamic mechanical analysis (DMA). Rectangular samples with dimensions of 30 × 10 × 5 mm were used. Samples were tested in shear oscillation mode in the temperature range from −50 °C to 180 °C (heating rate of 3 °C/min), and the frequency was 1 Hz and in the amplitude range of 0.1–2%. 

### 2.11. Thermomechanical Analysis and Safety Coefficient

The Linseis TMA PT instrument, manufactured in Selb, Germany, was used for thermomechanical analysis (TMA) tests. For the examination, rectangular cuboid samples that were about 2 cm tall (h) were generated. Each sample was cooled from 20 °C to −160 °C at a rate of 3 °C/min during the test and then heated to 50 °C (3 °C/min). Based on the TMA data collected during the cooling phase, which ranged from 22 °C (295 K) to −196 °C (77 K), the material shrinkage was calculated.

A material’s safety coefficient indicates how well it can withstand heat strains. Equation (1) was used for its calculation.

(1) $$k_{S}=\frac{\varepsilon_{77}}{{∆l}_{295-77}}$$

where ε_77_ is the tensile elongation at break at 77 K, %, and Δl_295−77_ is the relative expansion (shrinkage) of the material, cooling it from 295 to 77 K, %.

### 2.12. Scanning Electron Microscopy (SEM)

Rigid PU foam samples were analyzed by obtaining images via scanning electron microscopy (SEM) using a Vega Plus TS 5135 (Tescan, Brno, Czech Republic). The samples were cut in a frozen state and then fixed on a metallic support with carbon double-sided tape and sputtered with ~4 nm of gold in a high-vacuum sputter coater Leica EM SCD050 (Leica, Wien, Austria). 

## 3. Results and Discussion

### 3.1. Characterization of PCM

Phase-change materials (PCMs) are substances that absorb, store, and release thermal energy during the process of melting and crystallization. The characteristics of PCM selected for this study are listed in Table 2. The crystallization temperature is lower than the melting temperature, and the supercooling effect is about 5 °C. The melting latent heat is about the same as the crystallization latent heat. 

### 3.2. Density and Thermal Conductivity of PU Foams with Microencapsulated PCM

The following effects should be considered when introducing any dispersed filler into a rigid PU foam composition. Firstly, the viscosity of the composition component into which the filler is introduced increases (Table 3). This complicates the high-quality mixing of the polyol component with the isocyanate component, as well as the process of the normal growth and formation of rigid PU foam cells. If the critical concentration for each type of filler is exceeded, this can lead to the formation of defects in the cellular structure of the foam.

Secondly, any filler absorbs some of the exothermic heat of the urethane-formation reaction during the foaming of the rigid PU foam composition (Table 3). Accordingly, the part of the reaction heat spent on foaming the composition decreases. As a result, the foaming rate slows down, and the final density of the rigid PU foam may increase. In the case of PCM, this effect may be even more significant since some of the reaction heat will be spent not only on heating the dispersed filler but also on melting the PCM wax contained in it.

**Figure 1:**
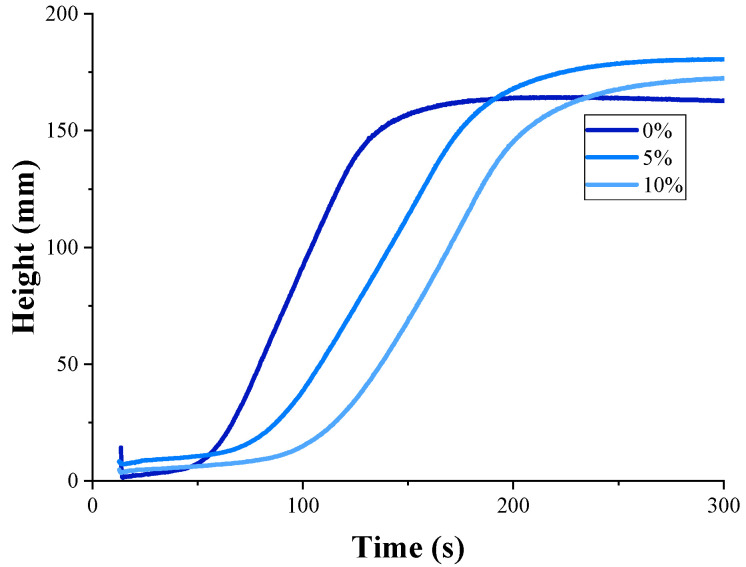
Foaming curves of the original and filled rigid PU foam composition.

The effect of slowing down the foaming rate of the rigid PU foam composition when introducing the microencapsulated PCM was ascertained during the standard cup test on the Foamat device. The foaming curves of the original rigid PU foam composition and the compositions containing 5 and 10% microencapsulated PCM are presented in Figure 1. The same trend was reported by Borreguero et al. [41].

This slowdown in the foaming rate resulted in a consistent increase in the foam density with an increase in the PCM content. The effect of a slight increase in density was observed for both the poured and sprayed rigid PU foam (Table 4). Thus, when introducing the maximum amount of PCM into the poured composition, the density increased by 9%. When introducing 5% PCM into the sprayed composition, the foam density increased by 6%. The addition of any kind of filler often results in an increase in the density [60,61,62], and this research is not an exception. 

However, such a slight increase in the density of the rigid PU foam did not practically affect the coefficient of thermal conductivity, due to the fact that the rigid PU foam filled with PCM retained a closed-cell structure (Table 4). Other authors, through the addition of PCM, observed an increase in the coefficient of thermal conductivity, but they added larger amounts of PCM [61,63]. 

### 3.3. Properties of Poured Rigid PU Foams

**Figure 2:**
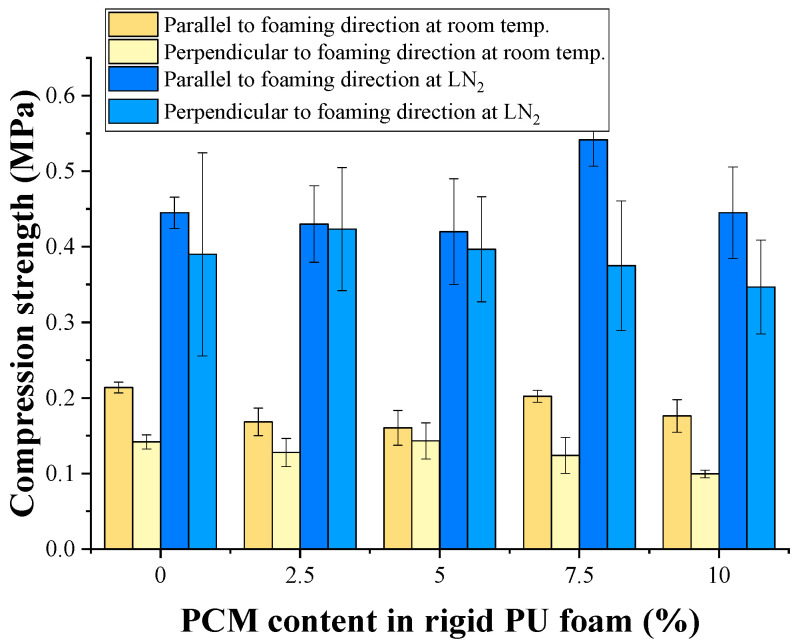
Compression strength of poured rigid PU foams.

The rigid PU foam compression strength in the foam rise direction at room and cryogenic (−196 °C) temperatures with the introduction of 2.5 and 5% PCM slightly decreased (Figure 2). However, with the introduction of 7.5%, when the foam plastic density increased to 40 kg/m^3^, the foam plastic strength at room temperature reached the level of the strength of the original rigid PU foam. Due to a slight increase in the density at a cryogenic temperature, it was even slightly higher than the strength of the original rigid PU foam.

The lowest compressive strength in the perpendicular direction at room and cryogenic temperatures was found in the rigid PU foam containing 10% PCM. The standard reason for such a decrease in the strength of foam plastic is the appearance of defects in its cellular structure. It is evident that from the point of view of compressive strength, 7.5% PCM content is critical, above which the strength of the foam plastic decreases due to an increase in the number of defects in the foam cellular structure.

**Figure 3:**
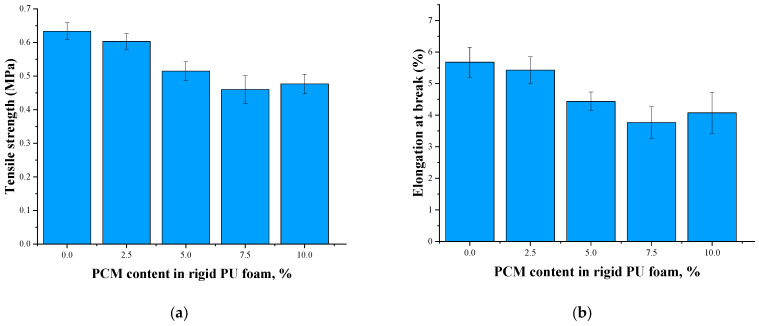
Tensile strength (**a**) and elongation at break (**b**) of rigid PU foams at an LN_2_ temperature.

**Figure 4:**
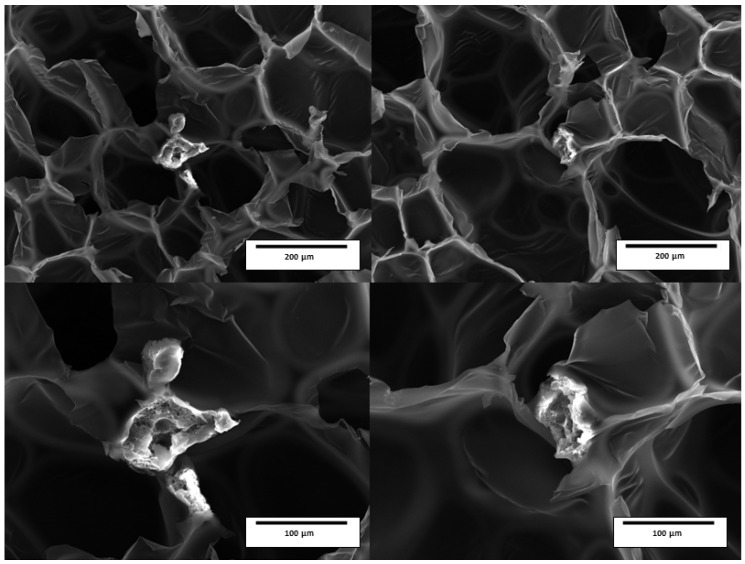
SEM images of rigid PU foams with PCM particles in the center of the images.

The gradual increase in the number of defects in the cellular structure with an increasing content of microencapsulated PCM is also the main reason for the decrease in the tensile strength and elongation at the break of the foam with an increasing filler content (Figure 3). The dimensions of the filler used (d = 100 μm) significantly exceeded the dimensions of the main load-bearing elements of the cellular structure of the low-density foam, such as the struts. Therefore, filler particles were only located in the nodes of the foam cells, as the SEM images proved (Figure 4). When the rigid PU foam is compressed, the effect of the filler in the nodes is insignificant. When the rigid PU foam is stretched, the filler in the cells’ nodes is more of a defect than a reinforcing element. Therefore, with an increase in the content of microencapsulated PCM, the tensile strength of the rigid PU foam, as well as the elongation at break, decreased.

**Figure 5:**
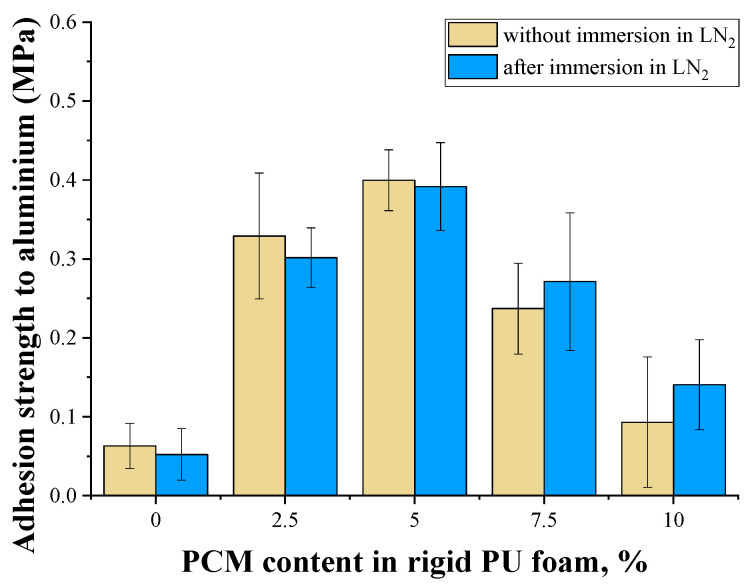
Adhesion to aluminum of poured rigid PU foams.

In contrast to the compressive and tensile strength, the adhesive strength of the rigid PU foam to aluminum increased with the introduction of the microencapsulated PCM. The highest adhesive strength was demonstrated for the foam containing 5% microencapsulated PCM (Figure 5). A possible reason for this effect was most likely the slowdown in the foam foaming process due to the previously described reasons when the filler was introduced. Foaming of the original PU foam occurred much more intensively than the filled rigid PU foam, most likely leading to higher initial thermal and shrinkage stresses. The latter obviously did not contribute to the emergence of a stronger adhesive bond between the foam and the aluminum substrate at the stage of contact layer formation.

With the introduction of a filler that absorbed additional heat due to the melting of the microencapsulated PCM wax, the foaming process was slower, which contributed to the formation of a stronger layer of rigid PU foam in contact with the metal substrate. The formation of such a stronger layer was also facilitated by a slight increase in the density of the foam with an increase in the PCM content to 5%. With a higher PCM content, the adhesive strength began to decrease. The reason for this decrease could be an increase in the viscosity of the rigid PU foam composition, which made it difficult to wet the metal surface in the initial stage of foam formation and a gradual increase in the number of defects in the foam cellular structure.

**Figure 6:**
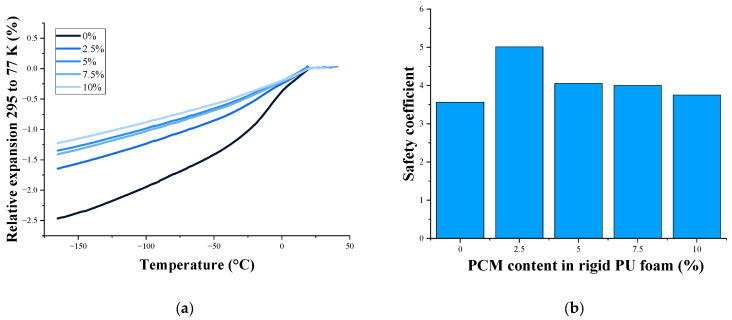
TMA curves (**a**) and safety coefficient (**b**) of rigid PU foams at an LN_2_ temperature.

Relative expansion decreased with an increasing PCM content (Figure 6a). The safety coefficient (Figure 6b) characterizes the material’s ability to tolerate thermal strains and absorb the cryo-shock. It is calculated using thermomechanical analysis and tensile test results. All rigid PU foams containing PCM had a higher safety coefficient than the original rigid PU foam. This indicated that PCM improves the materials’ ability to absorb the cryo-shock. With an increased PCM content, the safety coefficient decreased due to decreased elongation at break. 

### 3.4. Properties of Sprayed Rigid PU Foams

Taking into consideration all poured rigid PU foam results, it was decided to use only rigid PU foams with a 2.5 and 5 pbw PCM content for spraying tests. Those samples had a higher safety coefficient and adhesion than the original rigid PU foams and similar tension and compression properties. Also, the rigid PU composition with a higher PCM content significantly decreased the mechanical properties.

**Figure 7:**
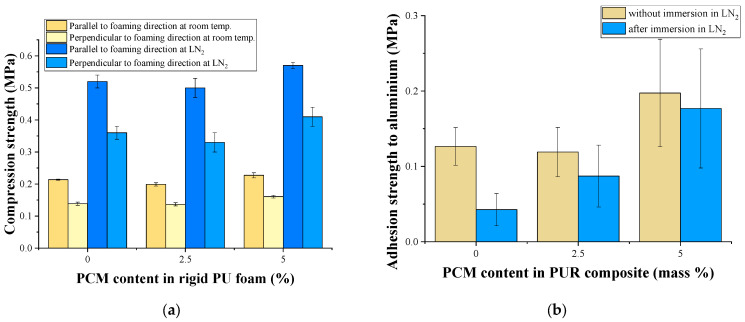
Compression strength (**a**) and adhesion strength (**b**) of sprayed rigid PU foams.

With the introduction of up to 5% microencapsulated PCM into the sprayed rigid PU foam composition, the compressive strength of the rigid PU foam plastic, both at room temperature and at −196 °C, remained practically unchanged (Figure 7a). In contrast to the compression strength, the adhesive strength of the foam plastic gradually increased with the introduction of PCM into the sprayed composition (Figure 7b). The rigid PU foam containing 5% PCM had the maximum adhesive strength. Moreover, with an increase in the PCM content, the difference in the initial adhesive strength of the foam and the adhesive strength after immersion in liquid nitrogen decreased. The reasons for the positive effect of PCM on the adhesive strength have already been discussed earlier. From the point of view of adhesion, a 5% PCM content is optimal for both poured and sprayed PU foams.

Introducing PCM in sprayed rigid PU foams lowered the tensile strength and elongation at break (Table 5). The same principle was observed for poured rigid PU foams but at a less pronounced rate. The tensile strength of the sprayed rigid PU foam without PCM was similar to the poured rigid PU foam, while sprayed rigid PU foams with PCM had lower tensile strengths. One of the reasons is that rigid PU foams with PCM had a more significant difference in density—sprayed foams had a lower density and, as a result, decreased tensile properties, as well. The second reason could be that defects in its cellular structure are more pronounced in sprayed foams.

Along with a decrease with the elongation at break, the safety coefficient of sprayed rigid PU foams also decreased (Table 3). Unlike the safety coefficient of poured rigid PU foams, the safety coefficient of sprayed rigid PU foams showed no increase after adding PCM. Lower safety coefficient values, together with lower adhesion strength values, mean that there is a need to work on the upscaling of rigid PU foams with PCM.

**Figure 8:**
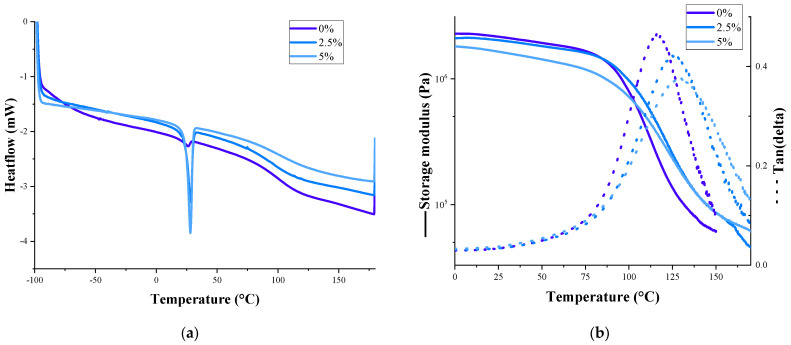
DSC (**a**) and DMA (**b**) curves of sprayed rigid PU foams (—— storage modulus, - - - tan(delta)).

The glass transition temperature and melting temperatures are determined using DSC. The. DSC curves of sprayed rigid PU foams show (Figure 8a) a distinct peak at 28 °C, for which the intensity increased with an increasing PCM content. This peak was the PCM melting temperature. The PCM melting temperature did not shift, and the peak intensity did not change after 50 heating/cooling cycles (Figure S1), showing that the encapsulated PCM is stable in rigid PU foams. The supercooling effect of PCM was 10 °C (Table 6). This indicates that the PCM will maintain its properties for at least 50 refills with a cryogenic liquid. The freezing temperature of the PCM in rigid PU foams was lower than pure PCM, which might indicate some interaction between the PCM and PU matrix. Qu et al. also obtained work with encapsulated PCM and obtained similar heat-of-fusion values as in our research [64]. Other authors obtained higher values but also worked with much higher PCM contents in the rigid PU foams [50,64,65,66].

The glass transition temperature, detected via DSC, slightly increased when PCM was added, from 95 °C (the sample without PCM) to 102 °C (the samples with 2.5% and 5% PCM). The DMA results (Figure 8b) show the same effect of an increasing temperature of the main transition (maximum of tan delta curve) with the PCM addition from 116 °C (0% PCM) to 125 °C (2.5% PCM) and 129 °C (5% PCM). Moreover, the main transition regions of the PU foams with PCM were broader than rigid PU foams without PCMs. This might be explained by PCM interaction with a rigid PU foam matrix—as PCMs are located only in nodes of rigid PU foam’s cell wall (see the SEM images in Figure 4), which mainly affects only those regions. This leads to localized changes in the PU structure and a less uniform foam structure overall.

The DMA storage modulus decreased with an increasing PCM content in the sprayed rigid PU foams. This trend was the same as the tension results, showing that the addition of PCM slightly reduced the mechanical properties of rigid PU foams.

## 4. Conclusions

This study investigated the influence of a microencapsulated phase-change material (PCM) on the properties of rigid polyurethane (PU) foams. Poured rigid PU foams were obtained with a 0–10% PCM content, after finding the limiting and optimal parameters (viscosity) of the composition-sprayed rigid PU foams obtained with 0–5% PCM content.

The central innovation of this study is the effect of PCM on the adhesion strength of the PU before and after the cryo-shock. In our work, it has been shown that at the optimal concentration of PCM, in our case 5%, the adhesion strength to aluminum increases from 0.1 MPa for unmodified PU foam to 0.4 MPa for the PU/PCM composite. The reason for this effect was the slowdown in the foam foaming process when the PCM was introduced. Adhesion to metal is a very important characteristic of good cryogenic insulation.

The second very important characteristic of cryogenic insulation—the safety coefficient—also increases for PU composites with PCM. For poured formulations, the maximum value is reached at a PCM concentration of 2.5% and it is 5, which characterizes the material as a good cryogenic insulation, since the limit values of the safety coefficient that must be exceeded are 3.

In addition, other characteristics of the PU/PCM composite were determined.

Increasing the PCM content in rigid PU foams leads to an increased density and decreased mechanical properties.

Further research should focus on the upscaling of the poured composition obtained by using the hand-mixing method, i.e., PU/PCM foam production with an industrial spraying machine. This is because in our preliminary tests with spraying compositions, the PCM effect on cryogenic properties was slightly lower than for poured compositions.

While the addition of PCM slightly reduced the mechanical properties of rigid PU foams, it improved the thermal performance and cryogenic resistance.

## 5. Patents

The patent application “Method for producing a rigid polyurethane foam composite for cryogenic insulation” has been submitted to the Patent Office of Latvia and registered under No. LVP2024000044.

## Figures and Tables

**Table 1:** Formulations of rigid PU foams.

	Weight, pbw	
	Poured	Sprayed
ETO_DEOA	15	15
ETO_DEG	10	10
NEO240	50	50
DEG	25	25
TCPP	15	15
Opteon 1100	31	31
Water	1.33	1.33
Polycat 203	0.2	4
Polycat 218	0.1	2
Dabco MB 20	0.05	0.2
Tegostab 84715	1.5	1.5
PCM	0–32.2	0−15.2
pMDI	148.8	148.8

**Table 2:** Characteristics of Crodatherm PCM.

Parameter	Value
Melting temperature, °C	29.3 ± 1.3
Freezing temperature, °C	24.5 ± 1.1
Heat of fusion (melting), J/g	178 ± 5
Heat of freezing (crystallization), J/g	−176 ± 8

**Table 3:** Foaming parameters of rigid PU foams with PCM and component A viscosity.

PCM in Rigid PU Foams, %	Start Time, s	Rise Time, s	Maximal Temperature, °C	Apparent Viscosity, mPa·s
0	49.7 ± 2.6	170.7 ± 3.7	53.6 ± 0.8	650 ± 40
2.5	62.0 ± 2.0	242.0 ± 3.0	52.2 ± 1.6	1600 ± 140
5	65.3 ± 0.0	232.6 ± 4.3	50.4 ± 1.2	3000 ± 340
7.5	79.7 ± 3.1	253.6 ± 4.5	50.0 ± 2.3	5200 ± 850
10	79.2 ± 1.7	270.0 ± 4.7	48.5 ± 1.1	6200 ± 1000

**Table 4:** Properties of poured and sprayed original and filled rigid PU foams.

PCM Content, %	Density, kg/m^3^		Coefficient of Thermal Conductivity, mW/m·K		Closed Cell, %	
	Poured	Sprayed	Poured	Sprayed	Poured	Sprayed
0	36.7 ± 0.7	34.2 ± 0.7	19.49 ± 0.10	18.64 ± 0.11	98.4 ± 0.3	95.7 ± 0.5
2.5	38.4 ± 0.5	34.8 ± 0.2	19.98 ± 0.12	18.97 ± 0.11	94.2 ± 0.3	95.6 ± 0.6
5	39.6 ± 0.5	36.2 ± 0.9	19.58 ± 0.11	18.78 ± 0.23	93.2 ± 0.1	95.3 ± 0.5
7.5	40.0 ± 1.5		19.81 ± 0.15		91.9 ± 1.3	
10	40.1 ± 0.9		19.63 ± 0.10		92.2 ± 0.5	

**Table 5:** Mechanical properties of sprayed rigid PU foams.

PCM Content, %	Tensile Strength, MPa	Elongation at Break, %	Relative Expansion 295 to 77 K, %	Safety Coefficient
0	0.60 ± 0.11	5.0 ± 0.9	1.57	3.2
2.5	0.46 ± 0.11	4.0 ± 0.9	1.30	3.0
5	0.48 ± 0.07	4.0 ± 0.4	1.37	2.9

**Table 6:** Thermal properties of rigid PU foams with PCM.

PCM Content, %	Melting Temperature, °C	Freezing Temperature, °C	Heat of Fusion, J/g	Glass Transition Temperature, °C	
				DSC	DMA
0				97.7 ± 1.8	116.0 ± 1.4
2.5	28.1 ± 0.8	18.1 ± 1.2	4.57 ± 0.5	101.9 ± 2.1	125.3 ± 2.1
5	28.0 ± 1.1	19.8 ± 0.9	6.51 ± 0.6	102.0 ± 2.0	129.2 ± 1.8

## Data Availability

The authors state that the presented data will be available on request by email.

## Supplementary Materials

The following supporting information can be downloaded at: https://www.mdpi.com/article/10.3390/polym17060729/s1, Figure S1: DSC curves of sprayed rigid PU foams with PCM contents of 2.5% (**a**) and 5% (**b**).
