# Supplementary figures and images for: Phase-Change Materials as Cryo-Shock Absorbers in Rigid Polyurethane Cryogenic Insulation Foams
Source: Polymers (Basel). 2025 Mar 10;17(6):729. doi: 10.3390/polym17060729 (PMC11944478; doi:10.3390/polym17060729)

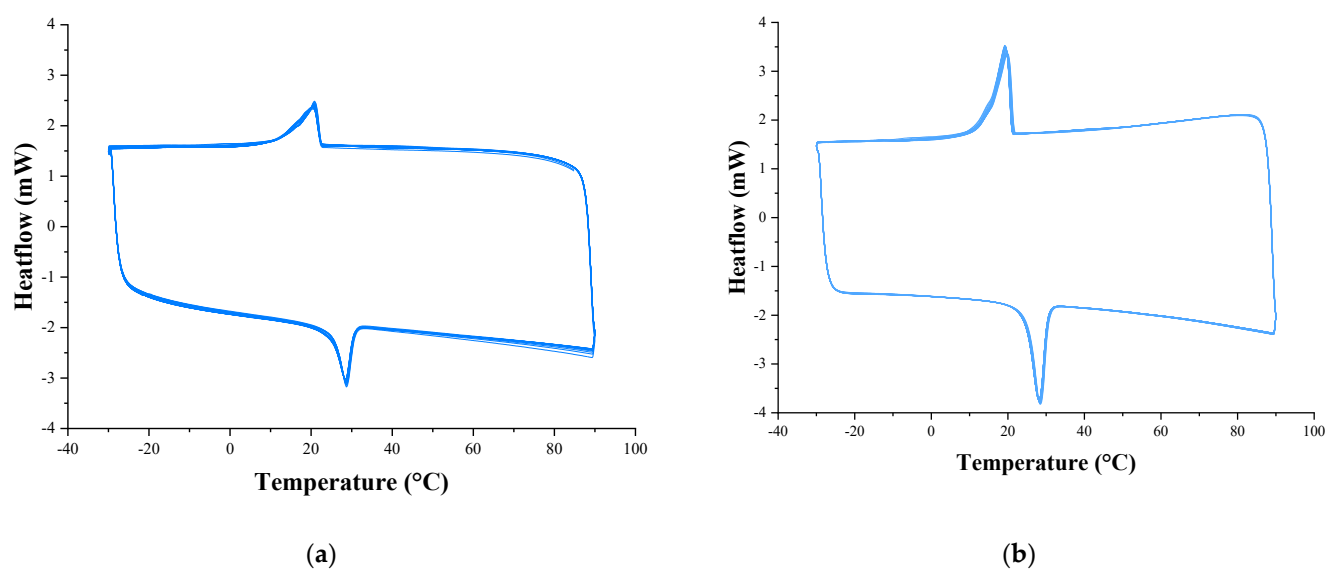

**Figure S1.** DSC curves of sprayed rigid PU foams with PCM content 2.5 % (a) and 5 % (b)

Supplement: Supplementary file 1 [file polymers-17-00729-s001.zip › polymers-3481245-supplementary.pdf]
